# 

**Published:** 1897-05

**Authors:** 


### BOOKS RECEIVED.

Atlas and Essentials of Gynecology. By Dr. Oscar Schaeffer, University of Heidelberg. Sixty-four full-page chromo-lithographic plates, comprising 159 figures; with descriptive text facing the plates, and over 300 pages of introduction and treatise, illustrated with wood-engravings. Duodecimo, muslin, $3.50, net. (Wood's Medical Hand Atlases, Volume V.)

Twentieth Century Practice. An International Encyclopedia of Modern Medical Science. By leading authorities of Europe and America. Edited by Thomas L. Stedman, M. D., New York City. In twenty volumes. Volume IX. Diseases of the Digestive Organs. New York: William Wood & Company. 1897.

Clinical Lessons on Nervous Diseases. By S. Weir Mitchell, M. D., LL. D., Edin., Member of the National Academy of Sciences; Honorary Fellow of the Royal Medico-Chirurgical Society of London. Handsome 12mo, 299 pages, with illustrations and two colored plates. Cloth, $2.50. Philadelphia and New York: Lea Brothers & Co., Publishers. 1897.

International Clinics. A Quarterly of Clinical Lectures on Medicine, Neurology, Surgery, Gynecology, Obstetrics, Ophthalmology, Laryngology, Pharyngology, Rhinology, Otology and Dermatology, and specially prepared articles on treatment. By professors and lecturers in the leading medical colleges of the United States, Germany, Austria, France, Great Britain and Canada. Edited by Judson Daland, M. D., (Univ. of Penna.) Philadelphia, Instructor in Clinical Medicine and Lecturer on Physical Diagnosis in the University of Pennsylvania; Assistant Physician to the Hospital of the University of Pennsylvania, etc.; Fellow of the College of Physicians of Philadelphia. J. Mitchell Bruce, M. D., F. R. C. P., London, England, Physician to, and Lecturer on, the Principles and Practice of Medicine at the Charing Cross Hospital. David W. Finlay, M. D., F. R. C. P., Aberdeen, Scotland, Professor of Practice of Medicine in the University of Aberdeen; Physician to, and Lecturer on, Clinical Medicine in the Aberdeen Royal Infirmary, etc. Volume I. Seventh series. April, 1897. Octavo, pp. xii.—344. Philadelphia: J. B. Lippincott Co. 1897.

Fourteenth Report of the State Board of Health of the State of New Hampshire from July 1, 1895, to November 1, 1896. Irving A. Watson, M. D., Secretary. Concord: Edward N. Pearson, Public Printer. 1897.

Lectures on the Treatment of Fibroid Tumors of the Uterus, Medical, electrical and surgical. By Franklin H. Martin, M. D., Professor of Gynecology Post-Graduate Medical School of Chicago; Surgeon Woman's Hospital of Chicago, etc., etc. Price, $1.00, net. Chicago: The W. T. Keener Co. 1897.

Annual Report of the Supervising Surgeon-General of the Marine Hospital Service of the United States. For the fiscal year 1896. Washington: Government Printing Office. 1896.

A Clinical, Pathological and Experimental Study of Fracture of the Lower End of the Radius. With Displacement of the Carpal Fragment Toward the Flexor or Anterior Surface of the Wrist. By John B. Roberts, A. M., M. D., Professor of Anatomy and Surgery in the Philadelphia Polyclinic; Professor of Surgery in the Woman's Medical College of Pennsylvania, etc. Octavo, pp. 76. Price, $1.00. With 33 illustrations. Philadelphia: P. Blakiston, Son & Co., 1012 Walnut street. 1897.

Diseases of the Ear, Nose and Throat and their Accessory Cavities. A Condensed Text-book. By Seth Scott Bishop, M. D., LL.D., Professor in the Chicago Post-Graduate Medical School and Hospital; Surgeon to the Illinois Charitable Eye and Ear Infirmary; Consulting Surgeon to the Illinois Masonic Orphans' Home and to the Silver Cross Hospital of Joliet. Illustrated with 100 colored lithographs and 168 additional illustrations. One volume, royal 8vo, pages xvi.—496. Extra cloth, $4.00 net; sheep or half-Russia, $5.00 net. Philadelphia: The F. A. Davis Co., Publishers, 1914 and 1916 Cherry street. New York: 117 W. Forty-second street. Chicago: 9 Lakeside building.

Warner's Pocket Medical Dictionary of Today. Comprising Pronunciation and Definition of 10,000 Essential Words and Terms used in Medicine and Associated Sciences. By William R. Warner. Philadelphia: William R. Warner & Co. 1897.

